# High prevalence of autoantibodies neutralizing type I IFNs in Japanese patients with hepatitis C

**DOI:** 10.70962/jhi.20250239

**Published:** 2026-09-16

**Authors:** Maki Taniguchi, Chiaki Tao, Takaki Asano, Miyuki Tsumura, Ko Ko, Hiroko Kumada, Takanori Utsumi, Kosuke Noma, Fumiaki Sakura, Kosuke Ashihara, Moe Tamaura, Yoko Mizoguchi, Osamu Ohara, Dusan Bogunovic, Paul Bastard, Jean-Laurent Casanova, Junko Tanaka, Masataka Tsuge, Kazuaki Chayama, Shiro Oka, Satoshi Okada

**Affiliations:** 1 Department of Pediatrics, Graduate School of Biomedical and Health Sciences, Hiroshima University, Hiroshima, Japan; 2 Department of Radiation Biophysics, Research Institute for Radiation Biology and Medicine, Hiroshima University, Hiroshima, Japan; 3 Graduate School of Biomedical and Health Sciences, Hiroshima University, Hiroshima, Japan; 4 Department of Applied Genomics, Kazusa DNA Research Institute, Chiba, Japan; 5 Laboratory of Human Genetics of Infectious Diseases, Necker Branch, INSERM, Paris, France; 6 Paris City University, Imagine Institute, Paris, France; 7 St. Giles Laboratory of Human Genetics of Infectious Diseases, Rockefeller Branch, The Rockefeller University, New York, NY, USA; 8 Department of Pediatrics, Necker Hospital for Sick Children, Assistance Publique–Hôpitaux de Paris, Paris, France; 9 Howard Hughes Medical Institute, New York, NY, USA; 10 Liver Center, Hiroshima University Hospital, Hiroshima, Japan; 11 Hiroshima Institute of Life Sciences, Hiroshima, Japan; 12 RIKEN Center for Integrative Medical Sciences, Yokohama, Japan; 13 Department of Gastroenterology, Graduate School of Biomedical and Health Sciences, Hiroshima University, Hiroshima, Japan; 14 Pediatric Haematology-Immunology and Rheumatology Unit, Necker Hospital for Sick Children, Assistance Publique – Hôpitaux de Paris, Paris, France; 15 Medical Policy Office, Hiroshima University, Hiroshima, Japan; 16 Department of Pediatrics, Columbia University Medical Center, NY, NY, USA; 17 Center for Genetic Errors of Immunity, Columbia University Medical Center, NY, NY, USA

## Abstract

Autoantibodies neutralizing type I IFNs (AAN-I-IFNs) are being identified as major, common, and global determinants of a growing range of severe viral diseases. We examined whether persistent hepatitis C virus (HCV) infection, with or without IFN-α therapy, could induce AAN-I-IFNs. We tested 2,573 HCV patients aged 1–95 years (1,115 treated with IFN-α, 1,458 untreated) and 1,000 healthy controls. AAN-INF-α2 prevalence was significantly higher in hepatitis C patients (2.7%) than in healthy controls (0.7%) and was higher still in those treated with IFN-α (3.9%) than in untreated patients (1.9%) (P = 0.0053). A longitudinal study of 15 IFN-α–treated patients with AAN-IFN-α2 revealed that only one had AAN-IFN-α2 before IFN-α therapy and that 12 patients developed AAN-IFN-α2 in the year following treatment. However, 80% eventually became AAN-IFN-α2 negative. These findings suggest that chronic HCV infection, perhaps due to the chronic production of endogenous IFN-α, and exogenous IFN-α treatment promote the development of AAN-IFN-α2.

## Teaser

The prevalence of autoantibodies neutralizing type I IFNs is significantly higher in Japanese patients with hepatitis C (2.7%) than in age-matched healthy controls (0.7%) and is even higher in those treated with IFN-α (3.9%) than in untreated individuals (1.9%).

## Introduction

Autoantibodies neutralizing type I IFNs (AAN-I-IFNs, such as IFN-α, IFN-β, IFN-ω, etc.) have been identified as an independent risk factor for life-threatening coronavirus disease 2019 (COVID-19) pneumonia caused by severe acute respiratory syndrome coronavirus 2 (SARS-CoV-2) (1). Bastard et al. discovered that 10.2% of patients with life-threatening COVID-19 pneumonia harbored AAN-I-IFNs (1). They also showed that AAN-I-IFNs were predominantly present in male patients (78.3%) in a cohort of patients with critical COVID-19 pneumonia. Subsequent studies replicated these findings of AAN-I-IFNs in patients with severe COVID-19 in various regions (2, 3, 4, 5, 6, 7, 8, 9, 10, 11, 12, 13, 14, 15, 16, 17, 18, 19, 20, 21, 22, 23, 24, 25, 26, 27, 28, 29, 30, 31, 32, 33, 34, 35, 36, 37, 38, 39, 40, 41, 42, 43, 44, 45, 46, 47, 48, 49, 50, 51, 52, 53, 54). AAN-I-IFNs were detected in about 20% of patients over 80 years of age with severe COVID-19 and in about 20% of patients of all ages who died from SARS-CoV-2 infection (5). In a cohort of uninfected individuals, the prevalence of AAN-I-IFNs was also higher in the elderly: 1% in those younger than 70 years, 2.3% in those aged between 70 and 80 years, and 6.3% in those older than 80 years (5). These results confirm that both age and sex are epidemiological risk factors for COVID-19. Furthermore, AAN-I-IFNs have been reported to be associated with adverse reactions to the yellow fever YFV-17D live-attenuated viral vaccine (55), severe influenza pneumonia (56), critical Middle East respiratory syndrome pneumonia (57), West Nile virus encephalitis (58), Powassan virus encephalitis, Usutu virus infection, Ross River virus disease (59), and tick-borne encephalitis (60).

The spontaneous production of pathogenic AAN-I-IFNs was first reported in 1981 (61, 62), but AAN-I-IFNs were initially discovered a few months earlier in a patient treated with IFN-β (63). AAN-I-IFNs are observed in patients treated with type I IFNs (63, 64, 65, 66) and in patients with systemic lupus erythematosus with excessive type I IFN production (67), thymoma (68), myasthenia gravis (69, 70), or hepatitis B (71). These patients may also present epidemiological risk factors for severe COVID-19 and other viral infections. Notably, some patients with chronic hepatitis C have AAN-I-IFNs. The underlying mechanisms of this phenomenon are thought to be induced by either the viral infection itself or by IFN treatment (72, 73, 74, 75). AAN-I-IFNs are frequently detected in patients treated with IFN-α, particularly in cases of poor response to treatment or relapses after IFN-α treatment (73, 74, 75, 76). However, those studies were conducted on relatively small samples (70–200 cases), so the details, including the underlying mechanisms, remain to be elucidated through large cohort studies.

Since the SARS-CoV-2 pandemic in 2020, several studies have reported that pre-existing chronic liver disease is associated with a higher risk of severe manifestations of COVID-19 (77, 78, 79, 80, 81, 82, 83). Marjot et al. calculated mortality rates for each category of the Child-Pugh classification in a study of 745 patients with chronic liver disease and found that the mortality associated with COVID-19 increased significantly with decreasing liver reserve function: 19% for Child-Pugh class A, 35% for class B, and 51% for class C, vs. 8% for non-cirrhotic patients during the period from March to July 2020 (81). Other studies have found a similar relationship between increased mortality and decreased liver reserve function (80, 83). The pathophysiological mechanisms underlying the higher rates of severe COVID-19 in patients with cirrhosis remain unclear, but the presence of AAN-I-IFNs is thought to be at least partly responsible. Greville et al. investigated the neutralizing activity of type I IFNs and the presence of autoantibodies in patients with decompensated cirrhosis in the absence of COVID-19 (84). That study further showed that while viral replication and infectivity remained consistent, levels of IFN-α2b–binding antibodies were high and levels of IFN-α2b activity were low, particularly in patients with a poor prognosis predicted by a high model for end-stage liver disease scores. These findings suggest that the presence of AAN-I-IFNs in patients with advanced cirrhosis may increase the risk of severe COVID-19. By contrast, the risk of severe disease and death from COVID-19 in patients with viral hepatitis without cirrhosis remains unclear (82). However, those positive for AAN-I-IFNs may have a higher risk of severe COVID-19, even in the absence of cirrhosis.

We therefore investigated the prevalence of AAN-I-IFNs in a large cohort of patients with hepatitis C, with the aim of determining whether chronic viral infection or exogenous IFN administration induced the production of these antibodies. We also investigated the association between clinical characteristics and AAN-I-IFN prevalence.

## Results

### Characteristics of the clinical manifestations of patients with hepatitis C

**Figure 1.**
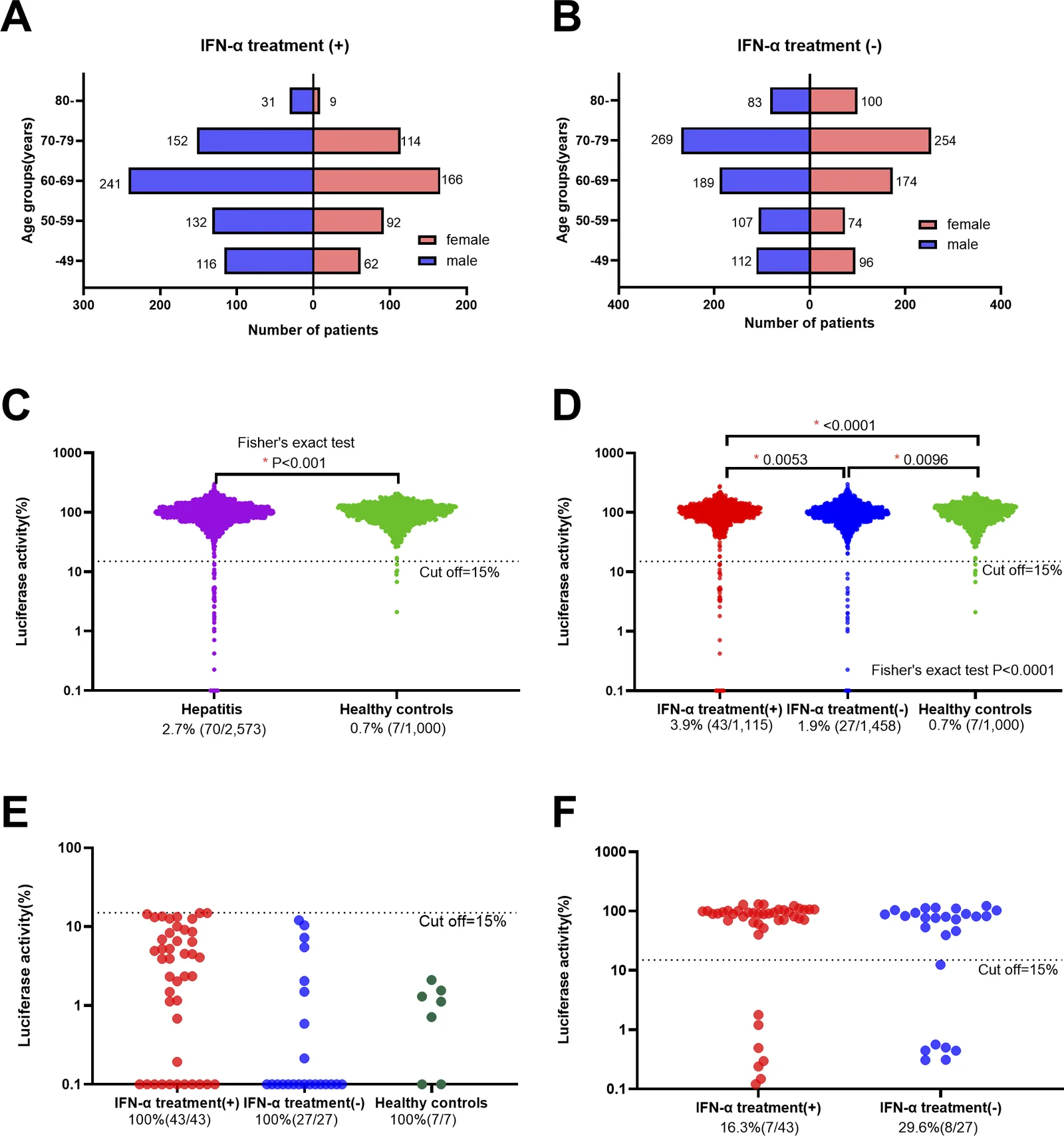
**IFN-α2 neutralization assays on serum from hepatitis patients and healthy controls. (A)** Age and sex distributions of hepatitis C patients treated with IFN-α (*n* = 1,115). The blue bar corresponds to male patients, and the pink bar corresponds to female patients. The median age was 64 years (range: 10–87 years); 60.3% of the patients were male, and 39.7% were female. **(B)** Age and sex distributions of hepatitis C patients without IFN-α treatment (*n* = 1,458). The blue bar corresponds to male patients, and the pink bar corresponds to female patients. The median age was 70 years (range: 1–95 years); 52.1% of the patients were male, and 47.9% were female. **(C)** Dot plot for the IFN-α2 (100 pg/ml) neutralization assay in 2,573 hepatitis C patients and 1,000 healthy controls. Samples were considered to have AAN-I-IFNs if luciferase activity was below 15%. The positivity rate was significantly higher in hepatitis C patients (70/2,573: 2.7%) than in healthy controls (7/1,000: 0.7%) (P < 0.001). **(D)** Dot plot of the neutralization assay in 1,115 hepatitis C patients treated with IFN-α, 1,458 patients without IFN-α, and 1,000 healthy controls. The positivity rate was significantly higher in hepatitis C patients treated with IFN-α (43/1,115: 3.9%) than in untreated hepatitis C patients (27/1,458: 1.9%) (P = 0.0053). **(E)** Neutralization assay using human cell–derived IFN-α2 (100 pg/ml) in 77 samples positive for AAN-IFN-α2 in the assay using *E. coli*–derived IFN-α2. Neutralizing activity was detected in all samples. **(F)** Neutralization assay using a higher concentration of IFN-α2 (10 ng/ml) in hepatitis C patients who had AAN-IFN-α2 (100 pg/ml). AAN-IFN-α2 (10 ng/ml) was detected in 7 of 43 patients with IFN-α and 8 of 27 patients without IFN-α, corresponding to 0.63% (7/1,115) and 0.55% (8/1,458) of the respective overall groups. The prevalence did not differ significantly between the two groups (P = 0.80).

We included 2,573 Japanese patients diagnosed with hepatitis C in this study. The detailed profiles of the participants are provided in Table 1. 1,115 patients were receiving IFN treatment (IFN treatment [+] group), and 1,458 were not (IFN-α treatment [−] group). The median age was 63 years (range: 10–87 years) in the IFN-α treatment (+) group, and 70 years (range: 1–95 years) in the IFN-α treatment (−) group. In the IFN-α treatment (+) group, 60.3% (*n* = 672) of patients were male and 39.7% (*n* = 443) were female. In the IFN-α treatment (−) group, 52.1% (*n* = 760) of patients were male and 47.9% (*n* = 698) were female. The proportion of male patients was significantly higher in the IFN-α treatment (+) group than in the IFN-α treatment (−) group (P < 0.0001) (Fig. 1, A and B). The following comorbid conditions were identified: HIV infection was present in 0.45% (*n* = 5) and HBV infection in 0.45% (*n* = 5) of patients in the IFN-α treatment (+) group and in 0.21% (*n* = 3) and 1.2% (*n* = 17) in the IFN-α treatment (−) group. Distribution of the *IL28B* single-nucleotide polymorphism (SNP) (rs8099917) in the IFN-α treatment (+) group was as follows: TT 73.3% (817/1,115), TG 21.0% (234/1,115), GG 1.3% (15/1,115), and no data available for 4.4% (49/1,115). In the IFN-α treatment (−) group, the distribution was: TT 61.0% (890/1,458), TG 17.0% (248/1,458), GG 1.0% (14/1,458), and no data available for 21.0% (306/1,458). Data on the duration of hepatitis were available for 74.7% (833/1,115) in the IFN-α treatment (+) group and 52.0% (758/1,458) in the IFN-α treatment (−) group. Among those patients, 79.6% (663/833) in the IFN-α treatment (+) group and 67.5% (512/758) in the IFN-α treatment (−) group had long-term disease of more than 5 years. In the IFN-α treatment (+) group, the type of IFN-α used and the time from IFN-α treatment initiation to serum collection varied, as shown in Table 2. In 661 cases (59.3%), the duration was less than 5 years, and 297 cases (26.6%) had a duration of less than 1 year. Conversely, 62 cases had a duration of 20 years or more. On classification according to the number of lines of treatment, 673 cases (60.3%) received only first-line IFN-α therapy, whereas some patients were on at least their fifth line of treatment, with a maximum of nine lines of treatment in some patients. Regarding the source of IFN-α2 preparations, 11.4% of patients (127/1,115) received native IFN-α2 alone, 66.9% (746/1,115) received recombinant IFN-α2 alone, and 21.7% (242/1,115) received both native and recombinant IFN-α2. Ribavirin was administered to 563 of 1,115 patients (50.5%) in the IFN-α treatment (+) group. The response to IFN treatment was as follows: sustained virological response (SVR) in 69.1% (771/1,115), non-SVR in 20.1% (224/1,115), and treatment discontinuation in 7.4% (83/1,115).

**Table 1.** Clinical data for hepatitis C patients and healthy controls

*N*	IFN-a treatment (+)	IFN-a treatment (-)	Healthy controls
	1,115	1,458	1,000
**Sex and age (years)**	​	​	​
Male	672	760	560
-<49	116	112	90
-50–59	132	107	95
-60–69	241	189	172
-70–79	152	269	161
->80	31	83	42
Female	443	698	440
-<49	62	96	63
-50–59	92	74	66
-60–69	166	174	134
-70–79	114	254	137
->80	9	100	40
**Other chronic viral infections**	​	​	​
HIV	5	3	0
HBV	5	17	0
-Chronic active infection	2	7	0
-Carrier	1	1	0
-Past infection	2	9	0
**IL-28B SNP(rs8099917) genotype**	​	​	N/A
TT (major)	817	890	​
TG (hetero)	234	248	​
GG (minor)	15	14	​
No Data	49	306	​
**Duration of hepatitis (years)**	​	​	N/A
<1	28	122	​
1–5	142	124	​
6–10	135	151	​
11–15	165	115	​
16–20	155	103	​
>20	208	143	​
**Response to IFN-α treatment**	​	N/A	N/A
SVR	771	​	​
Non-SVR	224	​	​
Discontinued	83	​	​
Other	10	​	​
No data	27	​	​

N/A, not applicable.

**Table 2.** Clinical data for hepatitis C patients treated with IFN-α

*n*	IFN-α treatment (+)
	1,115
**Time from initiation of IFN treatment to blood collection (years)**	​
<1	297
1–5	364
6–10	179
11–15	112
16–20	83
>20	18
Unknown	62
**No. of treatment lines**	​
First line	673
Second line	256
Third line	101
Fourth line	48
>Fifth line	37
Unknown	0
Prior treatment with IFNβ	119
**Total duration of IFN treatment (months)**	​
0–3	91
4–6	294
7–12	193
13–18	124
19–24	51
25–30	37
31–36	17
>37	45
Unknown	263
**Source of IFN-α2 preparations**	​
Native IFN-α2 alone	127
Recombinant IFN-α2 alone	746
Both native and recombinant IFN-α2	242
**Ribavirin co-administration**	​
Yes	563
No	552

As healthy controls, we included 1,000 individuals who underwent employee health examinations before the COVID-19 pandemic. These individuals were selected to yield age and sex distributions similar to those of the IFN-α treatment (+) and IFN-α treatment (−) hepatitis C patient groups.

### AAN-IFN-α2 (100 pg/ml) was frequently detected in patients with a history of IFN-α treatment, whereas AAN-IFN-α2 (10 ng/ml) was not

Autoantibodies neutralizing 100 pg/ml IFN-α2 were found in 2.7% (*n* = 70) of the 2,573 patients with hepatitis C, a value significantly higher than the 7/1,000 (0.7%) healthy controls with AAN-IFN-α2 (P < 0.001) (Fig. 1 C). The prevalence of AAN-IFN-α2 in hepatitis C patients was 3.9% (43/1,115) in the IFN-α treatment (+) group and 1.9% (27/1458) in the IFN-α treatment (−) group. Detection rates were significantly higher in the IFN-α treatment (+) group before (P = 0.0053) and after (P = 0.0007) adjustment for age and sex (Fig. 1 D). Thus, patients with hepatitis C have a higher frequency of AAN-IFN-α2 than healthy individuals, particularly if they are treated with IFN-α. This finding is not inconsistent with previous reports suggesting that both exposure to the virus itself and IFN treatment may be associated with an increased prevalence of AAN-I-IFNs based on observations in relatively small hepatitis C cohorts (70–200 cases) (72, 73, 74, 75).

We next examined the neutralizing activity against glycosylated IFN-α2 (100 pg/ml) produced in human cells (HEK293T) among individuals who tested positive for AAN-IFN-α2 (100 pg/ml) in each group. All individuals positive for AAN-IFN-α2 exhibited neutralizing activity against glycosylated IFN-α2 produced in human cells (Fig. 1 E).

Furthermore, we investigated the presence of AAN-IFN-α2 capable of neutralizing higher concentrations (10 ng/ml) in hepatitis C patients who tested positive for AAN-IFN-α2 (100 pg/ml), including 43 cases in the IFN-α treatment (+) group and 27 cases in the IFN-α treatment (−) group (Fig. 1 F). AAN-IFN-α2 (10 ng/ml) was detected in seven cases in the IFN-α treatment (+) group and eight cases in the IFN-α treatment (−) group, corresponding to 0.63% (7/1,115) and 0.55% (8/1,458) of the total cohort, respectively. No significant difference in the prevalence of AAN-IFN-α2 (10 ng/ml) was found between the two groups (P = 0.80).

The prevalence of AAN-IFN-α2 (100 pg/ml) was significantly higher in the IFN-α treatment (+) group than in the IFN-α treatment (−) group. At the same time, the prevalence of AAN-IFN-α2 (10 ng/ml) did not differ significantly between the two groups.

### No significant differences in the prevalence of AAN-IFN-ω or AAN-IFN-β among groups

**Figure 2.**
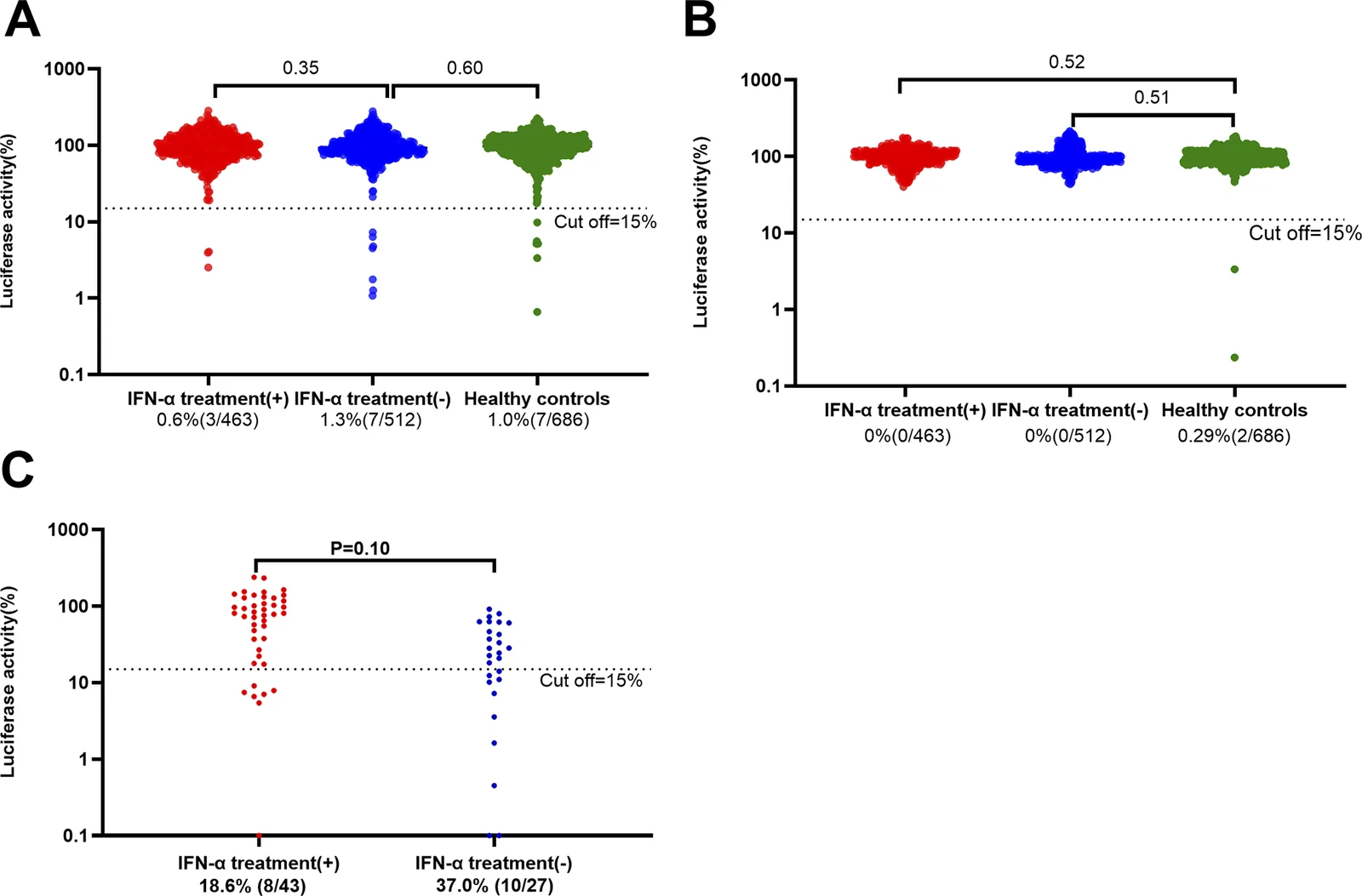
**Neutralization assays against IFN-ω and IFN-β in the selected subcohort. (A)** Neutralization assay against IFN-ω in a randomly selected subcohort representative of the overall cohort with respect to age, sex, and the prevalence of AAN-IFN-α2. The subcohort included 463 hepatitis C patients with IFN-α, 512 hepatitis C patients without IFN-α, and 686 healthy controls. The prevalence of AAN-IFN-ω was 0.6% (3/463), 1.3% (7/512), and 1.0% (7/686), respectively. No significant difference was observed between the IFN-α–treated and untreated groups or between the untreated group and healthy controls (P = 0.35 and P = 0.60, respectively). **(B)** Neutralization assay against IFN-β in the same subcohort. The prevalence of AAN-IFN-β was 0% (0/463), 0% (0/512), and 0.29% (2/686) in the IFN-α–treated group, untreated group, and healthy controls, respectively. No significant difference was observed between the IFN-α–treated group and healthy controls or between the untreated group and healthy controls (P = 0.52 and P = 0.51, respectively). **(C)** IFN-ω (100 pg/ml) neutralization assay in hepatitis C patients with AAN-IFN-α2 (100 pg/ml), with and without IFN-α treatment. The positivity rate was 18.6% (8/43) in the IFN-α treatment (+) group and 37.0% (10/27) in the IFN-α treatment (−) group (P = 0.10).

To evaluate the prevalence of AAN-IFN-ω and AAN-IFN-β, we selected a subcohort representative of the overall cohort with respect to age, sex, and the prevalence of AAN-IFN-α2. The subcohort comprised 463 patients in the IFN-α treatment (+) group, 512 patients in the IFN-α treatment (−) group, and 686 healthy controls. The prevalence of AAN-IFN-ω was 0.6% (3/463), 1.3% (7/512), and 1.0% (7/686), respectively. No significant differences were observed between the IFN-α treatment (+) group and the IFN-α treatment (−) group or between the IFN-α treatment (−) group and the healthy control group (P = 0.35 and P = 0.60, respectively) (Fig. 2 A). Similarly, the prevalence of AAN-IFN-β was 0% (0/463), 0% (0/512), and 0.29% (2/686), respectively. No significant differences were observed between the IFN-α treatment (+) group and healthy controls or between the IFN-α treatment (−) group and healthy controls (P = 0.52 and P = 0.51, respectively) (Fig. 2 B).

We subsequently identified and analyzed subgroups within the overall cohort that were expected to have a higher prevalence of AAN-IFN-ω and AAN-IFN-β. AAN-IFN-ω (100 pg/ml) was investigated in hepatitis C patients positive for AAN-IFN-α2 (100 pg/ml). Autoantibodies were detected in 18.6% (8/43) of patients in the IFN-α treatment (+) group and 37.0% (10/27) in the IFN-α treatment (−) group (P = 0.24) (Fig. 2 C and Table S1). These results suggest a tendency toward a high prevalence of AAN-IFN-ω in individuals positive for AAN-IFN-α2; however, no association was found with IFN-α treatment. A history of treatment with IFN-β was considered in 116 of the 1,115 patients in the IFN-α treatment (+) group. No patient with a history of IFN-β treatment tested positive for AAN-IFN-β.

Thus, in this randomly selected subcohort, we found no evidence of an increased prevalence of AAN-IFN-ω or AAN-IFN-β in patients with hepatitis C. These findings suggest that the association observed in our cohort is specific to AAN-IFN-α2 rather than reflecting a generalized increase in neutralizing autoantibodies against type I IFNs.

### The prevalence of AAN-IFN-α2 is higher in elderly men

**Figure 3.**
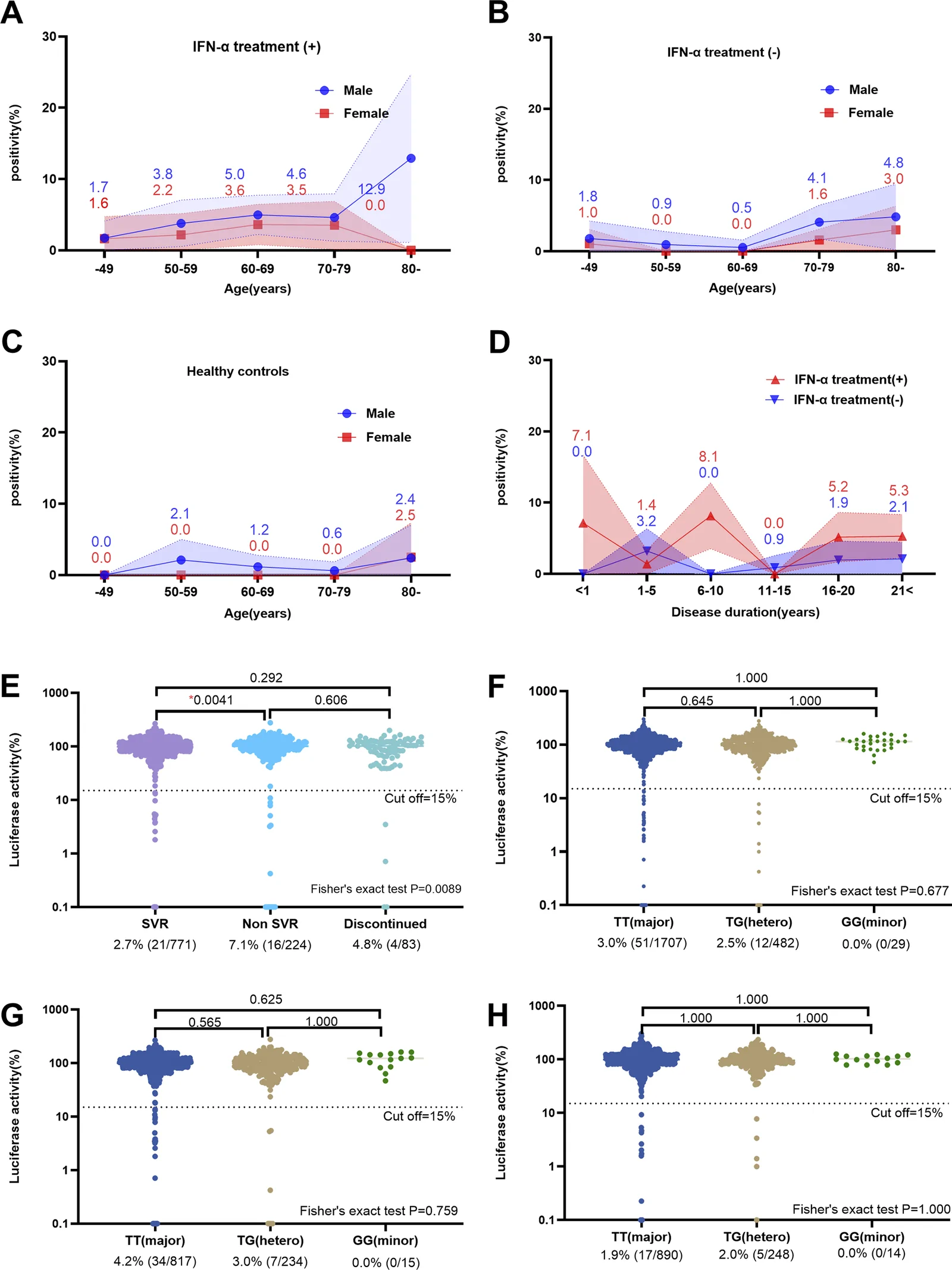
**AAN-I-IFN detection and clinical characteristics of the patients. (A)** AAN-IFN-α2 (100 pg/ml) positivity rates, by age and sex, in the IFN-α treatment (+) group. In men, autoantibodies were detected in 1.7% of individuals aged under 49 years, 3.8% aged in their 50s, 5.0% aged in their 60s, 4.6% aged in their 70s, and 12.9% aged 80 years or older. In women, the corresponding rates were 1.6%, 2.2%, 3.6%, 3.5%, and 0.0%, respectively. **(B)** AAN-IFN-α2 (100 pg/ml) positivity rates, by age and sex, in the IFN-α treatment (−) group. In men, autoantibodies were detected in 1.8% of individuals under 49 years, 0.9% aged in their 50s, 0.5% aged in their 60s, 4.1% aged in their 70s, and 4.8% aged 80 years or older. In women, the corresponding rates were 1.0%, 0.0%, 0.0%, 1.6%, and 3.0%, respectively. **(C)** AAN-IFN-α2 (100 pg/ml) positivity rates, by age and sex, in the healthy controls. In men, autoantibodies were detected in 0.0% of individuals aged under 49 years, 2.1% aged in their 50s, 1.2% aged in their 60s, 0.6% aged in their 70s, and 2.4% aged 80 years or older. In women, the corresponding rates were 0.0%, 0.0%, 0.0%, 0.0%, and 2.5%, respectively. **(D)** AAN-IFN-α2 (100 pg/ml) positivity rates by duration of hepatitis C infection. In the IFN-α treatment (+) group, the rates were 7.1% (duration <1 year), 1.4% (1–5 years), 8.1% (6–10 years), 0.0% (11–15 years), 5.2% (16–20 years), and 5.3% (>21 years). In the IFN-α treatment (−) group, the rates were 0.0%, 3.2%, 0.0%, 0.9%, and 1.9%, respectively. **(E)** AAN-IFN-α2 positivity rates by response to IFN-α treatment in hepatitis C patients. The rates were 2.7% (21/771) in SVR, 7.1% (16/224) in non-SVR, and 4.8% in patients in whom treatment was discontinued. The positivity rate was significantly higher in the non-SVR group than in the SVR group (P = 0.0041). **(F)** AAN-IFN-α2 positivity rates by SNP (rs8099917) genotype in hepatitis C patients were 3.0% (51/1709) for TT, 2.5% (12/482) for TG, and 0% (0/29) for GG (P = 0.677). **(G)** AAN-IFN-α2 positivity rates, by SNP (rs8099917) genotype, in the IFN-α treatment (+) group were 4.2% for TT, 3.0% for TG, and 0% for GG (P = 0.759). **(H)** AAN-IFN-α2 positivity rates, by SNP (rs8099917) genotype, in the IFN-α treatment (−) group were 1.9% for TT, 2.0% for TG, and 0% for GG (P = 1.000).

In the overall population of patients with hepatitis C, the prevalence of AAN-IFN-α2 was 3.4% (49/1432) in men and 1.8% (21/1141) in women (P = 0.014). In the IFN-α treatment (+) group, AAN-IFN-α2 were detected in 4.5% (30/672) of men and 2.9% (13/443) of women. There was a trend toward a higher rate of positivity in men, but this difference was not statistically significant (P = 0.208). In the IFN-α treatment (−) group, AAN-IFN-α2 were detected in 2.5% (19/760) of men and 1.2% (8/698) of women, but, again, this difference was not statistically significant (P = 0.078). The rate of positivity was significantly higher among hepatitis C patients than among controls in both men (3.4% [49/1432] vs. 1.1% [6/560], P = 0.0034) and women (1.8% [21/1141] vs. 0.2% [1/440], P = 0.014) (Table S2). These results suggest that male patients with hepatitis C have a higher risk of AAN-IFN-α2 than female patients. Among men in the IFN-α treatment (+) group, AAN-IFN-α2 were detected in 3.1% (11/354) of those under 65 years of age and 5.97% (19/318) in those aged 65 years or older (P = 0.092); the positivity rate increased with age from 1.7% for those under 49 years, 3.8–5.0% for those aged 50–79 years, and 12.9% for those aged 80 years and older. Among men in the IFN-α treatment (−) group, AAN-IFN-α2 were detected in 1.27% (4/314) of those under 65 years of age and 3.4% (15/446) of those aged 65 years or older (P = 0.097), 0.5–1.8% for those younger than 69 years, and 4.0% for those aged 70 years and older. Women displayed no age-related increase in the rate of AAN-IFN-α2 detection regardless of IFN-α treatment status (2.90% [7/241] in those under 65 years vs. 2.97% [6/202] in those aged 65 years or older, P = 1.00 for those with treatment; and 0.41% [1/241] vs. 1.5% [7/457], P = 0.27 for those without treatment) (Table S2 and Fig. 3, A–C). Overall, elderly patients with hepatitis C are at high risk of having AAN-IFN-α2, and this risk is particularly high for men. This finding is consistent with previous studies in the general population. In particular, the positivity rate exceeded 4% in patients over 60 years of age who had received IFN-α treatment, and in patients over 70 years of age in the IFN-α treatment (−) group. This finding is consistent with a previous report in which the median age at the onset of AAN-I-IFN production is about 63 years, based on a 35-year longitudinal study of 1,876 individuals with well-treated HIV infection (44). By contrast, our previous study of 622 Japanese COVID-19 patients based on the same method revealed an AAN-IFN-α2 (100 pg/ml) positivity rate of 0% in individuals younger than 49 years and 1.1% in women (25). However, the present study shows that 1.5% of hepatitis C patients younger than 49 years and 1.9% of women have AAN-IFN-α2. Therefore, particular attention should be paid to hepatitis C patients, as AAN-I-IFNs can be detected even in younger patients and women.

No consistent trend was observed for the relationship between rates of AAN-IFN-α2 detection and the duration of hepatitis in hepatitis C patients. The rates of AAN-IFN-α2 detection were as follows: 1.3% for a duration of <1 year, 2.3% for 1–5 years, 3.8% for 6–10 years, 0.36% for 11–15 years, 3.9% for 16–20 years, and 4.0% for over 21 years. There was no consistent trend in the relationship between rates of AAN-IFN-α2 detection and hepatitis duration in either the IFN-α treatment (+) group or the IFN-α treatment (−) group (Table S3 and Fig. 3 D). In the IFN-α treatment (+) group, there were five patients with HIV infection and five with HBV infection. In the IFN-α treatment (−) group, three patients had HIV infection, and 17 had HBV infection. None of these patients had AAN-IFN-α2. No consistent relationship was observed between AAN-IFN-α2 positivity and the duration of hepatitis or the presence of other chronic viral infections.

### The prevalence of AAN-IFN-α2 is higher in patients with a poor response to IFN-α treatment

In the IFN-α treatment (+) group, we also investigated the relationship between positivity for AAN-IFN-α2 and treatment efficacy. The positivity rate was 2.7% (21/771) for SVR and 7.1% (16/224) for non-SVR, which was significantly different (P = 0.0041) (Table S4 and Fig. 3 E). This finding is consistent with several previous reports showing a significantly higher rate of AAN-I-IFN detection in cases of non-SVR and relapse (73, 74, 75).

Individuals with the TT (major homozygous) genotype at the *IL28B* SNP (rs8099917) locus are known to have a better response to IFN-α treatment than those with TG (heterozygous) or GG (minor homozygous) genotypes (85). However, an analysis of AAN-IFN-α2 detection rates by *IL28B* SNP (rs8099917) in hepatitis patients showed these rates to be higher in TT than in TG patients, and higher in TG patients than in GG patients, but without these differences being significant (P = 0.677): TT 3.0% (51/1707), TG 2.5% (12/482), and GG 0% (0/29). Further, no significant differences in positivity rates were observed after stratification by IFN-α treatment: TT 4.2%, TG 3.0%, and GG 0% (P = 0.759) for the IFN-α treatment (+) group, and TT 1.9%, TG 2.0%, and GG 0% for the IFN-α treatment (−) group (P = 1.000) (Table S5 and Fig. 3, F–H). No consistent relationship was observed between positivity rates for AAN-IFN-α2 and the type of IFN-α used for treatment, time since initial IFN-α treatment, or number of lines of treatment (Table S6). Lastly, the prevalence of AAN-IFN-α2 was 3.9% (22/563) among patients who received ribavirin and 3.8% (21/552) among those who did not, with no significant difference between the two groups (P = 1.00). These findings suggest that AAN-IFN-α2 positivity is associated with a poor response to IFN-α treatment, whereas no significant association was observed with the IL28B SNP (rs8099917).

### The AAN-IFN-α2 positivity rate increased within 1 year of IFN-α treatment initiation

**Figure 4.**
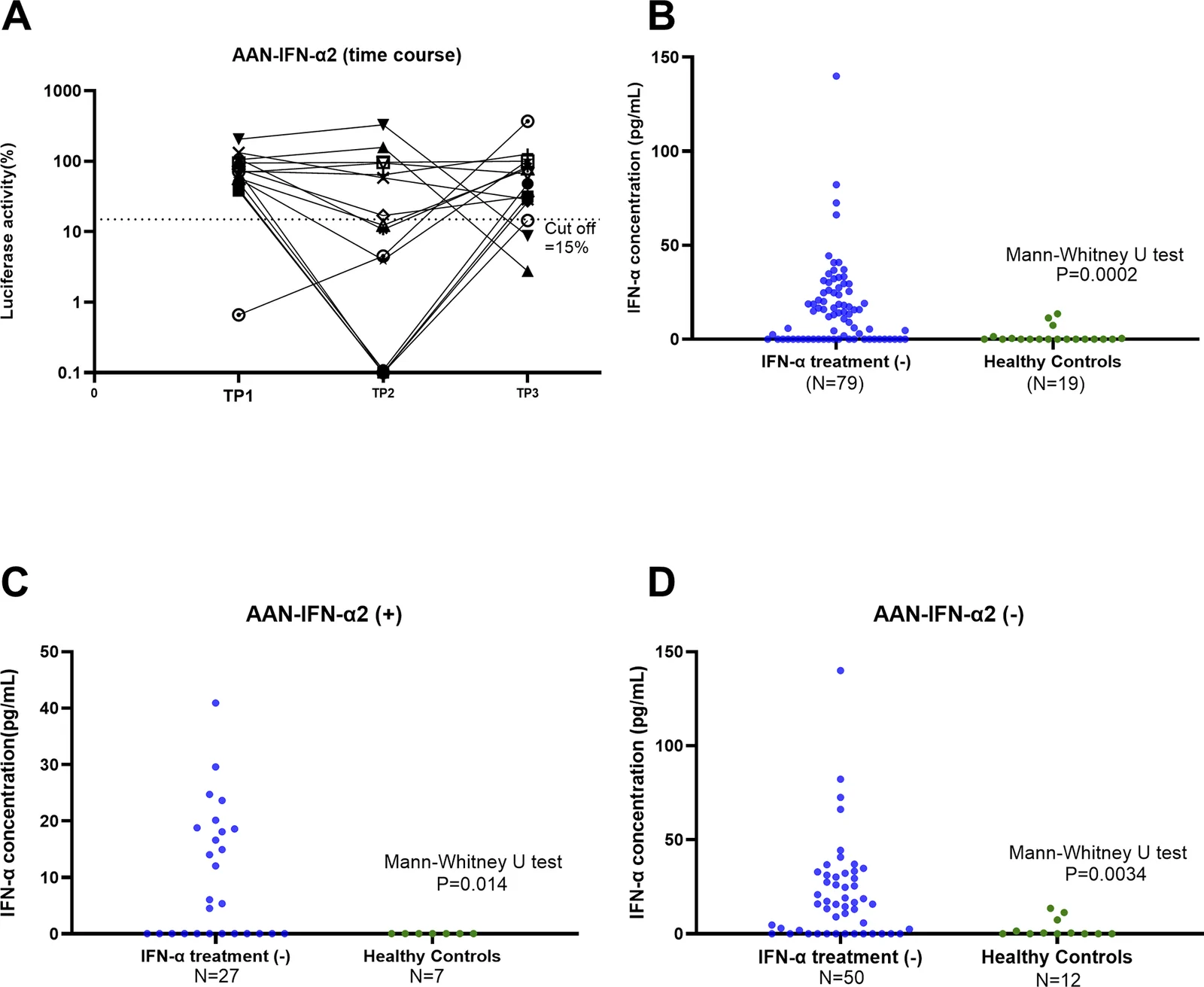
**Temporal changes in AAN-IFN-α2 neutralizing activity and serum IFN-α2 concentrations in patients with hepatitis C. (A)** Temporal dynamics of AAN-IFN-α2 (100 pg/ml) detection in 15 cases from the IFN-α treatment (+) group with AAN-IFN-α2. Neutralization assays were performed at three time points (TPs): TP1 (before IFN-α treatment initiation), TP2 (6 mo after treatment initiation), and TP3 (the most recent sample collected before the COVID-19 pandemic). At TP1, only one patient (6.7%) presented neutralizing activity. At TP2, eight cases (53.3%) tested positive. All patients tested positive at least once (data not shown), but 80% (12/15) had lost neutralizing activity by TP3. **(B–D)** Serum IFN-α2 concentrations were measured in 77 patients without IFN-α, including 27 AAN-IFN-α2–positive patients, and 19 healthy controls, including seven AAN-IFN-α2–positive individuals, using the ProQuantum Human IFN-α Immunoassay. The LOD was 0.05 pg/ml; values below the LOD were assigned a value of 0.05 pg/ml for graphical presentation and statistical analysis. **(B)** Serum IFN-α2 concentrations were higher in hepatitis C patients without IFN-α than in healthy controls (median, 13.7 pg/ml [range: <LOD to 140.0 pg/ml] vs. below the LOD [<LOD to 13.6 pg/ml]; P = 0.0002). **(C)** Among AAN-IFN-α2–positive individuals, serum IFN-α2 concentrations were higher in patients with hepatitis C than in healthy controls (median, 5.4 pg/ml [range: <LOD to 40.9 pg/ml] vs. below the LOD in all seven controls; P = 0.015). **(D)** Among AAN-IFN-α2–negative individuals, serum IFN-α2 concentrations were higher in patients with hepatitis C than in healthy controls (median, 15.8 pg/ml [range: <LOD to 140.0 pg/ml] vs. 0.23 pg/ml [<LOD to 13.6 pg/ml]; P = 0.0034).

The temporal dynamics of AAN-IFN-α2 production were investigated in AAN-positive individuals from the IFN-α treatment (+) group. Neutralizing activity was evaluated at three time points: (1) before the initiation of IFN-α treatment, (2) 6 mo after treatment initiation, and (3) in the most recent sample obtained before the COVID-19 pandemic. We analyzed 15 cases for which serum samples were available for all three time points. In two cases, IFN-β therapy was administered for 4–11 mo before a switch to IFN-α treatment. Only one of the 15 cases (6.7%) displayed anti–IFN-α2 neutralizing activity before treatment. This patient was 76 years old at the first time point. She had liver cancer, Alzheimer’s disease, and hyperlipidemia as comorbid conditions, but no history of autoimmune diseases or other severe infections. 6 mo after treatment initiation, eight cases (53.3%) tested positive for neutralizing activity. At least 12 of the initially negative cases (80.0%) had detectable autoantibodies within 1 year of treatment initiation, and all cases displayed neutralizing activity 98 mo after treatment initiation. However, in 12 of the 15 cases (80.0%), including the patient with IFN-α2–neutralizing activity before treatment, neutralizing activity was no longer detectable in the most recent samples collected after the completion of IFN-α therapy (Fig. 4 A and Table S7). The remaining three cases received IFN-α therapy as a first- to third-line treatment and presented persistent neutralizing activity at 38, 63, and 131 mo following the completion of final IFN-α treatment. One patient had a history of primary pulmonary leiomyosarcoma and a thyroid tumor. None of the three patients had a documented history of autoimmune disease, other chronic infections, or other malignancies; however, we could not evaluate subclinical autoimmunity before IFN-α treatment. Our results suggest that most AAN-IFN-α2–positive cases begin to produce these autoantibodies several months to 1 year after starting IFN-α treatment. Furthermore, in many cases, the AAN-IFN-α2 produced are not persistent and gradually disappear after the completion of treatment.

Only four of the 15 cases could be followed until the start of the COVID-19 pandemic in 2020. One of these four patients developed mild COVID-19, although the patient’s vaccination status was unknown. In contrast, all three patients with detectable AAN-IFN-α2 at time point three had died before the pandemic and were therefore not included among the four patients with available follow-up data during the COVID-19 pandemic. Their time point 3 samples were collected in 2012, 2018, and 2012, and they died in 2013, 2019, and 2012, respectively. No information on other viral infections was available for any of the 15 patients (Table S7).

### Patients with hepatitis C had higher concentrations of IFN-α2 than healthy controls

We measured serum IFN-α2 concentrations in 77 patients with hepatitis C who had not received IFN-α treatment, including 27 AAN-positive patients, and 19 healthy controls, including 7 AAN-positive individuals. IFN-α concentrations were quantified using the ProQuantum Human IFN-α Immunoassay, a quantitative PCR (qPCR)–based assay with a lower limit of detection (LOD) of 0.05 pg/ml.

The median IFN-α2 concentration was 13.7 pg/ml (range: less than the LOD to 140.0 pg/ml) in patients with hepatitis C who had not received IFN-α treatment, whereas it was below the LOD (range: <LOD to 13.6 pg/ml) in healthy controls (P = 0.0002) (Fig. 4 B). Among AAN-positive individuals, the concentrations were below the LOD in all seven healthy controls, consistent with our previous findings in patients with COVID-19 (25). In contrast, the median concentration among AAN-positive patients with hepatitis C was 5.4 pg/ml (range: <LOD to 40.9 pg/ml; P = 0.015) (Fig. 4 C). Among AAN-negative individuals, the median IFN-α2 concentration was 15.8 pg/ml (range: <LOD to 140.0 pg/ml) in patients with hepatitis C compared with 0.23 pg/ml (range: <LOD to 13.6 pg/ml) in healthy controls (P = 0.0034) (Fig. 4 D). These findings indicate that IFN-α2 concentrations are higher in patients with hepatitis C than in healthy controls. Furthermore, circulating IFN-α2 remained detectable in some AAN-positive patients with hepatitis C, suggesting that the presence of AANs does not uniformly result in undetectable circulating IFN-α2.

## Discussion

We investigated the prevalence of AAN-I-IFNs in Japanese patients with hepatitis C. We used serum samples collected before the COVID-19 pandemic, which we compared with samples from healthy controls. Cirrhosis (80, 81, 83), alcoholic liver disease (81), and nonalcoholic fatty liver disease (80, 82) were the only liver diseases considered to be risk factors for COVID-19 severity. This study provides the first large-scale assessment of AAN-I-IFN prevalence in patients with hepatitis C, a population in whom these autoantibodies may be clinically relevant to susceptibility to severe viral infections. The rate of AAN-IFN-α2 detection in patients with chronic hepatitis C was 2.7%, significantly higher than in healthy adults matched for ethnicity, age, and sex (0.7%). Moreover, among patients with hepatitis C, the rate of AAN-IFN-α2 detection was significantly higher in the IFN-α treatment group (3.9%) than in the group without IFN-α treatment (1.9%). Among patients with chronic hepatitis C, AAN-IFN-α2 was more prevalent in male patients and in older individuals for both the IFN-α treatment (+) and IFN-α treatment (−) groups.

Among the clinical characteristics examined in IFN-α–treated patients, we found that only the response to IFN-α therapy was associated with AAN-IFN-α2 positivity. The higher prevalence of AAN-IFN-α2 in untreated patients with hepatitis C than in healthy controls suggests an association between chronic hepatitis C and AAN-IFN-α2 positivity. However, the cross-sectional design of this study does not allow us to determine whether chronic hepatitis C virus (HCV) infection promotes the development of AAN-IFN-α2 or whether pre-existing AAN-IFN-α2 predisposes individuals to persistent HCV infection. Indeed, serum IFN-α2 concentrations were significantly higher in the IFN-α treatment (−) group than in healthy controls. Moreover, whereas serum IFN-α2 was below the LOD in all AAN-IFN-α2–positive healthy controls, detectable levels were observed in some AAN-IFN-α2–positive patients with hepatitis C. These findings suggest that endogenous IFN-α2 production may persist in chronic hepatitis C. We therefore hypothesized that the AAN-I-IFN positivity rate is higher in patients with a longer disease duration. However, no clear correlation was observed between disease duration and AAN-IFN-α2 positivity, regardless of IFN-α treatment status. In analyses of the relationship between the timing of AAN-IFN-α2 appearance and IFN-α2 treatment, only one patient tested positive before treatment initiation. Rather, most patients developed AAN-IFN-α2 within 1 year of IFN treatment initiation. AAN-IFN-α2 subsequently became undetectable in 80% of cases following the completion of IFN-α therapy. These findings are consistent with those of a previous study of 300 HIV-infected patients with HCV treated with Peg IFN-α, which reported the detection of AAN-IFN-α2 in three patients 6 mo after IFN-α treatment, with these antibodies persisting for more than 10 years in only one patient (44). We also found that the prevalence of AAN-IFN-α2 (100 pg/ml) was significantly higher in the IFN-α treatment (+) group than in the IFN-α treatment (−) group. However, the prevalence of AAN-IFN-α2 (10 ng/ml) did not differ significantly between the two groups. These findings suggest that AAN-I-IFNs may be induced by IFN treatment but at a titer that is not particularly high, with a decrease in levels after treatment discontinuation or hepatitis improvement.

In the selected subcohort, the prevalence of AAN-IFN-ω was 0.6% in the IFN-α treatment (+) group, 1.3% in the IFN-α treatment (−) group, and 1.0% in healthy controls, with no significant differences among the three groups. In contrast, the prevalence was 25.7% (18/70) in hepatitis C patients with AAN-IFN-α2 (100 pg/ml). This prevalence was comparably high with that of AAN-IFN-ω reported in AAN-IFN-α2–positive healthy individuals (43.0%) (5) and was unaffected by IFN-α treatment. These findings suggest that AAN-IFN-ω positivity may be more closely associated with the presence of AAN-IFN-α2 than with a history of IFN-α treatment. The prevalence of AAN-IFN-β was 0% in the IFN-α treatment (+) group, 0% in the IFN-α treatment (−) group, and 0.29% in healthy controls, with no significant differences among the three groups. Furthermore, none of the 116 patients with hepatitis C who had received IFN-β treatment tested positive for AAN-IFN-β when neutralizing activity was assessed using 10 ng/ml IFN-β. Thus, we found no evidence in this cohort that previous IFN-β treatment was associated with an increased prevalence of AAN-IFN-β. However, given the limited sample size, a possible effect of IFN-β treatment cannot be excluded. IFN-β treatment may be a viable option for combating COVID-19 infection in these AAN-I-IFN–positive individuals (86, 87, 88, 89, 90, 91, 92, 93, 94). Similarly, IFN-β may remain a potential therapeutic option for patients with AAN-IFN-α2, provided that neutralizing activity against IFN-β is absent.

This study has several limitations. First, there are missing data for the duration of hepatitis, the detailed course of the disease, and the degree of liver cirrhosis. In elderly cases, information about the final outcome of hepatitis C, the presence and severity of COVID-19, other infectious diseases, and other underlying conditions was not collected. Second, primary treatment chronology differed between patients who received IFN treatment and those who did not. IFNs were the cornerstone of treatment for chronic hepatitis C until the 2000s. However, direct-acting antivirals (DAAs) emerged in the 2010s and have been the mainstay of treatment since the mid-2010s (95). The IFN-α treatment (−) group was highly heterogeneous, including patients who were not candidates for IFN treatment and those treated solely with DAAs. Third, because of the limited number of positive healthy controls within each age- and sex-specific stratum, we were unable to perform sufficiently robust comparisons between patients with hepatitis C and healthy controls after simultaneous stratification by both age and sex. Fourth, IFN-α2–binding antibodies were not assessed by ELISA or other binding assays; therefore, the relationship between binding antibody levels and neutralizing activity could not be evaluated.

Despite these limitations, this study provides a large-scale assessment of the prevalence and clinical correlates of AAN-I-IFNs in Japanese patients with hepatitis C. Because COVID-19 outcomes were unavailable for most participants, the clinical impact of these autoantibodies on the risk or severity of COVID-19 could not be evaluated directly. Nevertheless, our findings suggest an association between hepatitis C infection and the presence of AAN-IFNs. AAN-IFN-α2 positivity was associated with a poor response to IFN-α treatment. However, because detailed information on liver disease progression and long-term outcomes was incomplete, the relationship between AAN-I-IFNs and the natural course of hepatitis C could not be determined. This study also identified the following as risk factors for AAN-I-IFN production: advanced age, male sex, treatment with IFN-α, and an insufficient response to IFN-α treatment.

HCV infection induces the production of type I IFNs in the liver (96). The production of AAN-I-IFNs in patients with HCV may therefore be linked to the virus-induced production of endogenous type I IFNs. Moreover, the prevalence of AAN-IFN-α2 increases following IFN-α treatment in these patients. It is, therefore, reasonable to suggest that endogenous and/or exogenous exposure to type I IFNs can trigger the development of these AAN-I-IFNs. The notion that viral infections create conditions conducive to the development of AAN-I-IFNs was also proposed in a recent Swiss study (44). Based on previous and our findings, we hypothesize that prolonged exposure to endogenous type I IFNs during chronic viral infection may contribute to the development of AAN-I-IFNs in susceptible individuals. However, the direction of causality remains uncertain and should be examined in longitudinal studies using samples obtained before infection or before the establishment of chronic disease.

## Materials and methods

### Patient samples, data, and ethics

Patients with hepatitis C who visited the Department of Gastroenterology and Metabolism at Hiroshima University Hospital between April 1992 and May 2020 were enrolled. We collected available serum samples for the investigation. Patients were stratified into two groups based on their history of IFN-α treatment up to the time of serum collection: those who had received treatment (IFN-α treatment [+]) and those who had not (IFN-α treatment [−]). In the IFN-α treatment (+) group, patients were treated with any type of IFN-α (e.g., pegylated IFN-α, consensus IFN-α). The IFN-α treatment (−) group included patients who received no treatment, symptomatic treatment only, hepatoprotective drug treatment only, and antiviral drug treatment. In this group, serum samples were mostly collected at the initial visit. Two samples in this group were excluded due to sample quality issues.

For hepatitis C patients, we evaluated clinical information, such as *IL28B* SNP (rs8099917) genotype, duration of hepatitis C, and response to IFN-α treatment in the IFN-α treatment (+) group. *IL28B* SNP (rs8099917) genotype is known to be associated with responsiveness to IFN-α and ribavirin treatments, with a significantly poorer response in TG (heterozygous) and GG (minor homozygous) individuals than in TT (major homozygous) individuals (85). The response to IFN-α therapy was classified as “SVR,” characterized by undetectable HCV viremia 24 wk after the end of therapy, “non-SVR” in patients who did not achieve SVR, and “discontinued” if IFN-α treatment was stopped, principally due to adverse events.

Serum samples from a cohort of 1,000 healthy subjects were used as controls. This cohort consisted of 1,000 workers who underwent an employee health check in 2017, before the COVID-19 pandemic. The composition of the cohort was adjusted to match the age and sex ratios of the IFN-α treatment (+) and IFN-α treatment (−) groups. The study was conducted on an existing sample meeting the criteria for inclusion following an opt-out presentation. Approval was obtained from the Hiroshima University Institutional Review Board (E2020-2011-08).

### Detection of anti-cytokine autoantibodies: Luciferase reporter assay

In this study, the neutralization of IFN-α2, IFN-ω, and IFN-β was assessed in an IFN-stimulated response element (ISRE) reporter assay. Paul et al. previously reported that patients with AAN-IFN-α2 also display neutralizing activity against all other IFN-α subtypes (5). We therefore assessed the neutralization of 100 pg/ml IFN-α2 in this study. We further evaluated neutralizing activity against a higher concentration of IFN-α2 (10 ng/ml) in patients who tested positive for AAN-IFN-α2 at 100 pg/ml. Neutralizing activity against IFN-ω (100 pg/ml) and IFN-β (10 ng/ml) was also assessed in a randomly selected subcohort. The minimum sample size was calculated using Cochran’s formula with a finite population correction, and participants were randomly selected to obtain a subcohort representative of the overall cohort with respect to age, sex, and the prevalence of AAN-IFN-α2. In addition, we analyzed subgroups expected to have a higher prevalence of these autoantibodies. AAN-IFN-ω was assessed in patients with hepatitis C who tested positive for AAN-IFN-α2 at 100 pg/ml, whereas AAN-IFN-β was assessed in patients with a history of IFN-β treatment.

Neutralizing activity was evaluated by measuring luciferase reporter activity as previously described (25). Briefly, HEK293T cells were used to seed 96-well plates at a density of 2.5 × 10^4^ cells/well in 100 μl medium and incubated at 37°C for 16 h. We then used X-tremeGene9 transfection reagent (Roche Diagnostics) to transfect the cells with a luciferase reporter plasmid containing the firefly luciferase gene under the control of the human ISRE promoter in the pGL4.45 backbone and a control reporter plasmid, pRL-SV40, for normalization. After 24 h of incubation, we added 10% serum from participants diluted in Dulbecco’s modified Eagle medium (Thermo Fisher Scientific) supplemented with 2% HyClone fetal bovine serum (GE Healthcare Life Sciences). We stimulated cells at 37°C with recombinant human (rh) IFN-α2 (No.: 130-093-874 [*Escherichia coli*]; Miltenyi Biotec; No.: HZ-1066; Proteintech [human 293 cell]), rhIFN-ω (No.: BMS304; eBioscience) or rhIFN-β (No.: 514005; BioLegend) for 8 h at cytokine concentrations of 100 pg/ml (rhIFN-α2, rhIFN-ω) or 10 ng/ml (rhIFN-α2, rhIFN-β). Finally, cells were lysed for 20 min at room temperature, and luciferase activity was measured with an Enspire plate reader (PerkinElmer Life Sciences) and the dual-luciferase reporter assay system (Promega).

The neutralizing activity of autoantibodies was assessed as follows. Firefly luciferase activity values were normalized against *Renilla* luciferase activity values. The resulting values were then normalized against the median induction levels for non-neutralizing samples from healthy controls (*n* = 5) tested on the same day. Luciferase activity (%) was calculated as follows: luciferase activity (%) = (Pf/Pr) ÷(Cf/Cr)×100, where Pf = firefly luciferase activity of the patient, Pr = *Renilla* luciferase activity of the patient, Cf = median firefly luciferase activity of healthy controls, and Cr = median *Renilla* luciferase activity of healthy controls. Based on previous findings (5), samples were considered to have neutralizing activity if they had a luciferase activity below 15% of control levels. In our analysis, the mean luciferase activity of IFN-α2 was 136.4 in the IFN-α treatment (+) group, 159.5 in the IFN-α treatment (−) group, and 58.1 in the healthy controls, indicating a significantly lower level of activity in the healthy controls. However, the serum samples from healthy controls were stored at a higher temperature, which may have contributed to their lower levels of activity. We therefore normalized the mean activity values of each group to 100 for comparative analysis.

### Measurement of IFN-α2 concentration

Serum IFN-α2 concentrations were measured using the ProQuantum Human IFN-α Immunoassay Kit (Invitrogen) according to the manufacturer’s instructions. Briefly, serum samples were diluted 10-fold with assay dilution buffer. 5 μl of the diluted samples were mixed with an equal volume of antibody–conjugate mixture and incubated for 1 h at room temperature. Following incubation, 40 μl of qPCR reaction mixture was added to each sample. Quantification was performed using a StepOnePlus Real-Time PCR System (Applied Biosystems), and data were analyzed with StepOne Software. The measured IFN-α2 concentrations were multiplied by 10 to calculate the original in vivo concentrations.

### Statistics

Statistical analysis was performed with JMP software (SAS Institute). We used nonparametric Kruskal–Wallis tests to compare the age distribution, the Mann–Whitney U test to compare the IFN-α2 distribution across different groups, and Fisher’s exact test to compare categorical variables, such as rates of AAN-I-IFN positivity. We assessed the effects of age and sex on the presence of AAN-I-IFNs in a multivariate logistic regression analysis. A two-tailed P value below 0.05 was considered statistically significant.

### Online supplemental material

Table S1 shows the prevalence of AAN-IFN-α2 (10 ng/ml) and/or AAN-IFN-ω (100 pg/ml) in AAN-IFN-α2 (100 pg/ml)–positive hepatitis C cases. Table S2 describes the prevalence of AAN-IFN-α2 (100 pg/ml) by age and sex. Table S3 describes the prevalence of AAN-IFN-α2 by duration of hepatitis C. Table S4 describes the prevalence of AAN-IFN-α2 by treatment response to IFN-α2. Table S5 describes the prevalence of AAN-IFN-α2 by SNP. Table S6 describes the prevalence of AAN-IFN-α2 by IFN-α treatment. Table S7 shows the temporal dynamics of AAN-IFN-α2 as determined by luciferase activity and clinical information for the AAN-IFN-α2–positive case in the IFN-α treatment (+) group.

## Supplementary Material

Table S1 shows the prevalence of AAN-IFN-α2 (10 ng/ml) and/or AAN-IFN-ω (100 pg/ml) in AAN-IFN-α2 (100 pg/ml)–positive hepatitis C cases.

Table S2 describes the prevalence of AAN-IFN-α2 (100 pg/ml) by age and sex.

Table S3 describes the prevalence of AAN-IFN-α2 by duration of hepatitis C.

Table S4 describes the prevalence of AAN-IFN-α2 by treatment response to IFN-α2.

Table S5 describes the prevalence of AAN-IFN-α2 by SNP.

Table S6 describes the prevalence of AAN-IFN-α2 by IFN-α treatment.

Table S7 shows the temporal dynamics of AAN-IFN-α2 as determined by luciferase activity and clinical information for the AAN-IFN-α2–positive case in the IFN-α treatment (+) group.

## Data Availability

The data on which this study is based are not publicly available due to patient privacy issues. They are available from the corresponding author upon reasonable request.
